# Infection prevention and control training and capacity building during the Ebola epidemic in Guinea

**DOI:** 10.1371/journal.pone.0193291

**Published:** 2018-02-28

**Authors:** Heidi M. Soeters, Lamine Koivogui, Lindsey de Beer, Candice Y. Johnson, Dianka Diaby, Abdoulaye Ouedraogo, Fatoumata Touré, Fodé Ousmane Bangoura, Michelle A. Chang, Nora Chea, Ellen M. Dotson, Alyssa Finlay, David Fitter, Mary J. Hamel, Carmen Hazim, Maribeth Larzelere, Benjamin J. Park, Alexander K. Rowe, Angela M. Thompson-Paul, Anthony Twyman, Moumié Barry, Godlove Ntaw, Alpha Oumar Diallo

**Affiliations:** 1 Centers for Disease Control and Prevention, Atlanta, United States of America; 2 Institut National de Santé Publique, Conakry, Guinea; 3 Catholic Relief Services, Conakry, Guinea; 4 World Health Organization, Conakry, Guinea; 5 Hôpital National Donka, Conakry, Guinea; 6 Guinea Ministry of Health and Public Hygiene, Conakry, Guinea; Katholieke Universiteit Leuven Rega Institute for Medical Research, BELGIUM

## Abstract

**Background:**

During the 2014–2016 Ebola epidemic in West Africa, a key epidemiological feature was disease transmission within healthcare facilities, indicating a need for infection prevention and control (IPC) training and support.

**Methods:**

IPC training was provided to frontline healthcare workers (HCW) in healthcare facilities that were not Ebola treatment units, as well as to IPC trainers and IPC supervisors placed in healthcare facilities. Trainings included both didactic and hands-on components, and were assessed using pre-tests, post-tests and practical evaluations. We calculated median percent increase in knowledge.

**Results:**

From October–December 2014, 20 IPC courses trained 1,625 Guineans: 1,521 HCW, 55 IPC trainers, and 49 IPC supervisors. Median test scores increased 40% (interquartile range [IQR]: 19–86%) among HCW, 15% (IQR: 8–33%) among IPC trainers, and 21% (IQR: 15–30%) among IPC supervisors (all P<0.0001) to post-test scores of 83%, 93%, and 93%, respectively.

**Conclusions:**

IPC training resulted in clear improvements in knowledge and was feasible in a public health emergency setting. This method of IPC training addressed a high demand among HCW. Valuable lessons were learned to facilitate expansion of IPC training to other prefectures; this model may be considered when responding to other large outbreaks.

## Introduction

During the 2014–2016 Ebola virus disease (Ebola) epidemic in West Africa, a key epidemiological feature was disease transmission within healthcare facilities, especially among healthcare workers (HCWs) [1–4]. As of January 20, 2016, the Guinea Ministry of Health and Public Hygiene reported 196 (5.8%) cases among HCWs of the 3,351 laboratory-confirmed cases of Ebola [5, 6]. HCWs in healthcare facilities that were not Ebola treatment units (ETUs) were at higher risk of exposure to Ebola, as they were responsible for: 1) triaging all incoming patients, who had unknown Ebola infectious status at presentation; and 2) attending to suspect Ebola cases awaiting transfer to an ETU [2, 4]. Numerous opportunities for transmission in healthcare facilities were present, due to insufficient infection prevention and control (IPC) measures.

IPC measures, which may include training and support, supplies, and systems for triage and isolation, can help protect HCWs and prevent Ebola transmission in healthcare facilities [3, 4]. To support IPC efforts in Guinea, a comprehensive strategy was designed to include training, supervision, provision of personal protective equipment (PPE) and other IPC supplies, and monitoring and evaluation. This report presents the implementation and results from early IPC training efforts from October–December 2014, at the height of the Ebola epidemic, and highlights possible key strategies to rapidly implement IPC in healthcare facilities within the context of a massive public health response.

## Materials and methods

IPC training materials were developed by staff from the Guinea Ministry of Health and Public Hygiene, the World Health Organization (WHO), Guinea’s *Institut National de Santé Publique* (INSP), and the U.S. Centers for Disease Control and Prevention (CDC), and were based upon Ebola IPC guidance from WHO and CDC.

All IPC trainings were conducted by Catholic Relief Services, in conjunction with approximately 5–7 staff members from the Guinea Ministry of Health and Public Hygiene, INSP, WHO, and CDC. These trainings included didactic and hands-on training on Ebola disease transmission, patient triage, donning and doffing PPE, safely cleaning bodily fluid spills, medical waste management, and preparation of chlorinated water solutions for hand washing and disinfection. Trainings alternated between didactic (55% of training time) and hands-on (45%) segments.

From October–December 2014, two IPC courses were developed and conducted for non-ETU settings. The first course targeted trainers of the curriculum and IPC supervisors, who were physicians, nurses, or other health professionals assigned to oversee IPC activities at a particular facility or group of facilities. This course was a 3- or 4-day training, and also included practice conducting rapid IPC needs assessments for non-ETU facilities, using a standard and thorough quality control checklist. These assessments measured aspects of IPC including facility and administrative controls such as proper triage and isolation, and behavioral aspects such as handwashing and correct use of PPE; and identified areas for improvement. For the first course, trainings were held in U.S. Peace Corps facilities in Conakry as the U.S. Peace Corps had evacuated its volunteers, or in school classrooms in the prefecture of N’Zérékoré.

**Fig 1:**
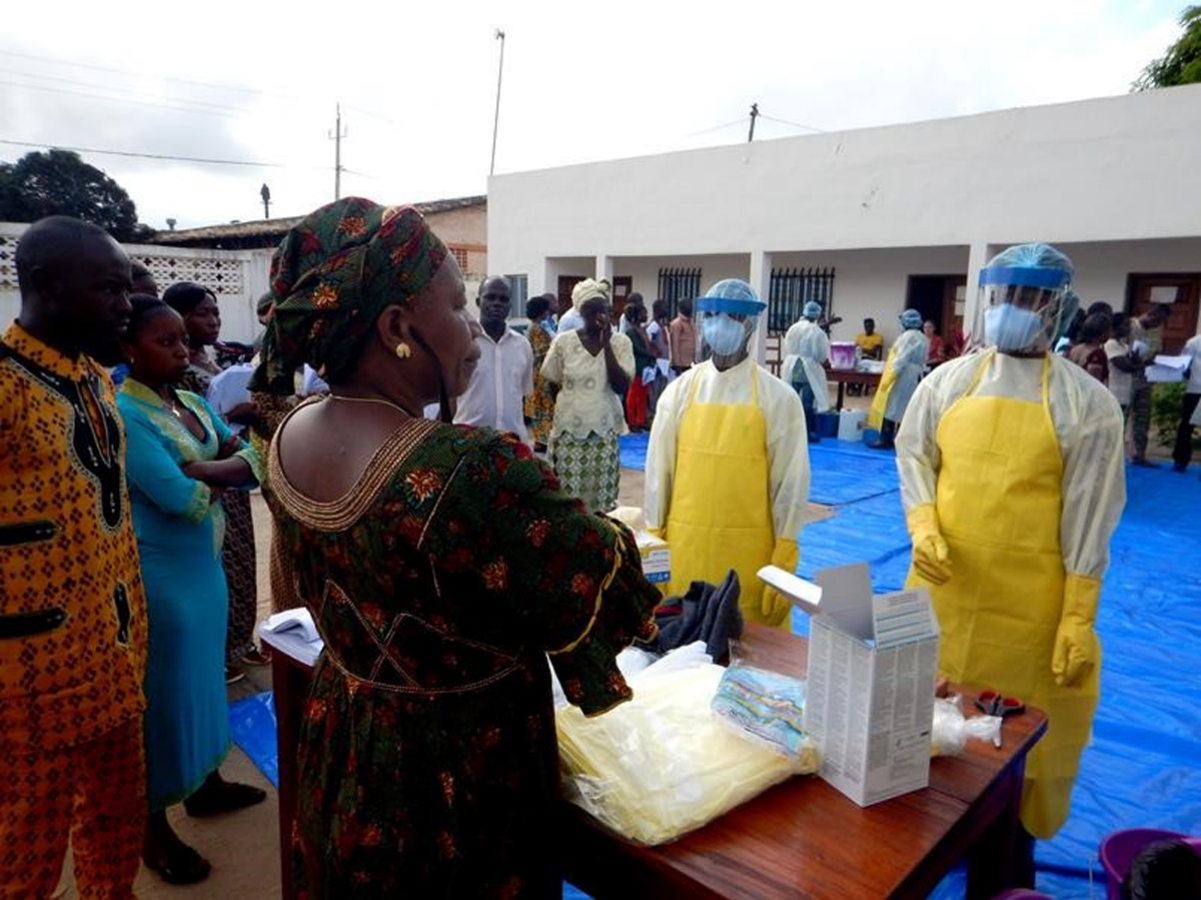
Personal protective equipment practice at infection prevention and control training—N’Zérékoré, Guinea, November 2014.

The second IPC course targeted frontline HCWs and was a condensed 2-day course that focused on rapidly delivering the IPC guidance and allowing HCWs the opportunity to practice through hands-on simulations (Fig 1). IPC training for frontline HCWs from non-ETU healthcare facilities was held in three prefectures in the forest region of southeastern Guinea (N’Zérékoré, Macenta, and Kérouané), where the highest numbers of Ebola-infected HCWs were at the time. Eligible HCW training participants included all persons working in healthcare facilities, including clinical, janitorial, and support staff. A broad definition of healthcare facility was used, including hospitals, clinics, public and private facilities, pharmacies, and informal clinics, as patient care is provided in each of these settings. Trainings were conducted with a ratio of approximately two IPC trainers per 16 HCWs. Trainings were held in school classrooms, as the Guinean government had closed schools to prevent Ebola transmission. Training participants received breakfast, lunch, coffee breaks, and a per diem. Attendance from a given healthcare facility was staggered to maintain continuous operations.

Upon arrival at the IPC training sites, trainers and participants were first asked to wash hands; then were screened with a non-contact infrared thermometer and asked Ebola-specific screening questions, per Guinea Ministry of Health and Public Hygiene protocol. These practices both protected the health and safety of all persons involved in the training and provided an example of how to implement triage procedures at a health facility.

To assess changes in knowledge associated with the training, a 30-question, multiple-choice test regarding Ebola and IPC practices (S1 Appendix) was developed by staff from the Guinea Ministry of Health and Public Hygiene, WHO, INSP, and CDC to broadly reflect the content of the training materials, and was administered to all participants at the beginning and end of each IPC training. As the test was developed for emergency response, it had not previously been validated. After the post-test, correct answers were reviewed with participants. Additionally, participants completed two practical evaluations at the end of training: one on donning and doffing PPE, and one on the preparation of chlorinated water solutions. These evaluations were scored as “acceptable,” “needs improvement,” or “unacceptable” by an expert trainer. Paired Wilcoxon signed rank tests were used to compare pre-test and post-test scores using SAS 9.3 (Cary, NC). This analysis of anonymized training data was determined not to require CDC Institutional Review Board review. The individuals in Fig 1 have given written informed consent to publish this image.

## Results

From October 8 –December 16, 2014, 1,625 Guineans participated in 20 IPC courses (Table 1). Three training sessions trained 49 IPC supervisors from Conakry, N’Zérékoré and Macenta, while three trainings in Conakry trained 55 IPC trainers. Thirty-four of the IPC trainers were hired, and they subsequently trained 1,521 frontline HCWs: 1,047 in N’Zérékoré, 355 in Macenta, and 119 in Kérouané.

**Table 1:** Summary of infection prevention and control training participants—Guinea, October–December 2014.

	Frontline HCWs	IPC supervisors	IPC trainers^*^	Total
Number of trainings	14	3	3	20
Number of HCWs trained	1,521	49	55	1,625
Gender, N (%)				
Male	718 (47)	40 (89)	34 (92)	792 (49)
Female	803 (53)	5 (11)	3 (8)	811 (51)
Unknown	0	4	18	22
Health sector, N (%)				
Public	1230 (81)	25 (74)	n/a	1,255 (81)
Private	282 (19)	9 (26)	n/a	291 (19)
Other	9 (1)	0 (0)	n/a	9 (1)
Missing	0	15	55	70
Job role, N (%)				
Doctor	107 (7)	3 (9)	55 (100)	165 (10)
Nurse	153 (10)	1 (3)	0 (0)	154 (10)
Pharmacist	20 (1)	0 (0)	0 (0)	20 (1)
Laboratorian	108 (7)	21 (62)	0 (0)	129 (8)
Health technician**	775 (51)	5 (15)	0 (0)	780 (48)
Midwife	130 (9)	1 (3)	0 (0)	131 (8)
Administrator	21 (1)	1 (3)	0 (0)	22 (1)
Student	33 (2)	0 (0)	0 (0)	33 (2)
Cleaner	94 (6)	0 (0)	0 (0)	94 (6)
Other	80 (5)	2 (6)	0 (0)	82 (1)
Missing	0	15	0	15

Abbreviations: HCW, healthcare workers; IPC, infection prevention and control; n/a, not available.

^*^ Health sector was not collected for all IPC trainers.

**Health technician is a broad category that includes nurse aides, hygiene workers, and surveillance workers.

Among the 49 IPC supervisors, 89% were male, three-quarters were employed in the public sector, and the majority were laboratorians (Table 1). All of the IPC trainers were doctors by design, and 92% were male. Of the 1,521 trained frontline HCWs, 803 (53%) were female; 1,230 (81%) were employed by public healthcare facilities, 282 (19%) worked in the private health sector, and 9 (1%) worked in other settings (Table 1). Participant job roles were as follows: 107 (7%) doctors, 153 (10%) nurses, 20 (1%) pharmacists, 108 (7%) laboratorians, 775 (51%) health technicians, 130 (9%) midwives, 21 (1%) administrators, 33 (2%) students, 94 (6%) cleaners, and 80 (5%) other types of jobs.

Among the IPC supervisors and IPC trainers, pre-test knowledge of Ebola symptoms and transmission was high. Both groups had a median pre-training score of 23 out of 30 (77%); median post-training score increased to 28 out of 30 (93%) (Table 2). Among a subset of 10 IPC supervisors who were re-tested four weeks after their initial training, knowledge retention was high; all scored ≥90%. For the practical evaluation of donning and doffing PPE, 97% of IPC supervisors and 83% of IPC trainers received a score indicating their practice was “acceptable,” 3% and 17% respectively “need improvement,” and none were “unacceptable.” During the preparation of chlorinated water solutions practical evaluation, approximately 80% of IPC supervisors and trainers received a score of “acceptable,” 17–18% “need improvement,” and only one in each group was “unacceptable.”

**Table 2:** Summary of infection prevention and control training scores—Guinea, October–December 2014.

	Frontline HCW^*^	IPC supervisors^†^	IPC trainers^‡^
Pre-test score, median (IQR)^§^	17 (12–21)	23 (20–27)	23 (20–24)
Post-test score, median (IQR)^§^	25 (22–28)	28 (27–29)	28 (27–29)
Increase in test score, median (IQR)^§^	7 (4–11)	4 (2–6)	5 (4–7)
Wilcoxon signed rank p-value	<0.0001	<0.0001	<0.0001
Percent increase in test score, median (IQR)	40% (19–86)	15% (8–33)	21% (15–30)
Donning/doffing PPE, n (%)			
Acceptable	815 (70)	33 (97)	44 (83)
Needs improvement	329 (28)	1 (3)	9 (17)
Unacceptable	14 (1)	0 (0)	0 (0)
Preparation of chlorinated water, n (%)			
Acceptable	911 (80)	27 (79)	43 (81)
Needs improvement	216 (19)	6 (18)	9 (17)
Unacceptable	16 (1)	1 (3)	1 (2)

Abbreviations: HCW, healthcare workers; IPC, infection prevention and control; IQR, interquartile range; PPE, personal protective equipment.

^*^ Of the 1,521 frontline HCW, 1,188 had an available pre-test score, 1,189 had an available post-test score, 1,176 had both pre-test and post-test scores, 1,158 had a donning/doffing PPE score, and 1,143 had a preparation of chlorinated water score.

^†^ Of the 49 IPC supervisors, 44 had an available pre-test score, 45 had an available post-test score, 44 had both pre-test and post-test scores, 34 had a donning/doffing PPE score, and 34 had a preparation of chlorinated water score.

^‡^ Of the 55 IPC trainers, 16 had an available pre-test score, 53 had an available post-test score, 16 had both pre-test and post-test scores, 53 had a donning/doffing PPE score, and 53 had a preparation of chlorinated water score.

^§^ Pre-test and post-test scores are out of a total of 30 questions.

For frontline HCWs, of the 30 pre-training questions, the median number of correct answers was 17 (57%, interquartile range [IQR]: 12–21), which increased to a median post-training score of 25 correct answers (83%, IQR: 22–28). Among 1,176 HCWs with both pre- and post-training test scores available, the median increase in the number of correct responses between the pre- and post-training test was 7 (IQR: 4–11; 40% median increase; Wilcoxon signed rank p-value <0.0001). For the practical evaluation of donning and doffing PPE, 70% of HCWs received a score indicating their practice was “acceptable,” 28% “needs improvement,” and 1% “unacceptable.” Most participants experienced donning and doffing PPE for the first time during the IPC training. During the preparation of chlorinated water solutions practical evaluation, 80% of training participants received a score of “acceptable,” 19% “needs improvement,” and 1% “unacceptable.”

Pre-training test scores varied according to frontline HCW profession, with doctors and administrators having a median of 21 out of 30 (IQR: 15–25), as compared to a median of 17 (IQR: 12–21) among the other professions (p-value <0.0001). Doctors also had the highest post-training test scores, with a median of 27 (IQR: 25–29), as compared to a median of 25 (IQR: 22–27) among the other professions (p-value <0.0001).

## Discussion

In the 2014–2016 Ebola epidemic, large impacts on the healthcare system resulted from Ebola transmission in healthcare settings due to gaps in IPC practices. This paper describes an early rollout of IPC trainings during the height of the epidemic, and demonstrates that large-scale IPC training efforts in the setting of a public health emergency may be feasible and effective.

We found that overall general baseline knowledge of Ebola among both IPC trainers, IPC supervisors, and frontline HCWs was relatively high. This could reflect previous Ebola trainings or exposure to Ebola-related public health messages, which were highly prevalent in the community. However, baseline knowledge of IPC practices appeared low, particularly among frontline HCWs. These findings are consistent with the high frequency of HCW infections, suggesting a low level of IPC training or supplies prior to the outbreak or infections occurring outside of the healthcare setting. Persons identified as IPC trainers or supervisors had a higher baseline knowledge level, as would have been expected given that they were qualified to be selected as trainers or supervisors. Knowledge was assessed using a multiple-choice test format, which could result in some correct answers by guessing, instead of other methods that may have better evaluated baseline knowledge but were more difficult and time-consuming to administer. Additionally, as the Ebola epidemic caused unprecedented IPC challenges, pre-validated training materials and tests were not available and had to be created specifically for these IPC trainings.

Post-training test and practical evaluation scores indicated a substantial increase in knowledge and ability, and among the subset of IPC supervisors for whom follow-up data were available, short-term knowledge retention also was high. These data suggest that even in the chaotic environment of an active public health crisis, IPC trainings can be conducted and result in immediate improvements in knowledge and practical skills. However, more follow-up would be required to know whether the attendees of the trainings experienced sustained knowledge retention and a reduction in their risk for Ebola following the training, and to confirm whether the IPC trainings were a key component to reducing that risk and preventing Ebola infections and deaths among HCWs. Regarding PPE use, each participant practiced donning and doffing PPE at least twice during the training, and was also given a handout clearly outlining the donning/doffing steps for future practice or use.

Despite the apparent success in the trainings, there were a number of lessons learned. We faced numerous logistical challenges such as finding clean training spaces, a lack of available training space once schools re-opened, arranging a consistent power supply, and obtaining and transporting training materials. Additionally, it was difficult to identify all frontline HCWs in the public, private, and informal healthcare facilities to include in the trainings, as no updated master list of HCWs existed. There was a limited pool of health professionals qualified to be trained as IPC trainers or IPC supervisors, due to a general lack of IPC knowledge. Additionally, at most HCW training sessions, up to three times as many HCWs arrived as were on the scheduled list. While this complicated the logistics and often delayed the start of the training day, it reflected local community acceptance and the strong demand for this type of practical training. Only in Kérouané did training teams encounter any resistance and therefore training was cut short. Combining frontline HCWs with different roles within the same training session prompted productive discussion and a common understanding that dedication to IPC principles by each member of the team would be required to protect all patients and HCWs within a facility against Ebola. Some necessary modifications to the training plan included holding training sessions specifically for cleaners or traditional birth attendants who could not read or write, providing training sessions in local languages, and including separate PPE practical sessions specifically for burial team volunteers.

Following the trainings, IPC supervisors were subsequently assigned to provide IPC supervision and support to healthcare facilities and HCWs. In addition, many of the professionals who developed and conducted the IPC trainings also conducted IPC assessments at individual healthcare facilities according to requests or demonstrated needs during the Ebola response.

This report summarizes early IPC training efforts that were conceived and implemented during an acute public health emergency. Given the need to train massive numbers of HCWs, it was not initially possible to conduct trainings in work environments, which would more readily allow HCWs to identify gaps in their IPC practices and settings. The described IPC trainings did incorporate hands-on exercises and evaluations, as well as time to discuss HCW experiences and concerns, providing training participants with some opportunity to practice and tailor the training to their personal circumstances, but conducting trainings within work environments could have led to more effective learning.

These IPC training efforts were necessary because an IPC infrastructure, which is a more ideal approach, did not exist and takes time to develop. In Guinea, progress toward sustainable IPC systems continues. National IPC policies are being developed and improved, and key implementation partners are providing critical mentorship and continued training. Focusing on IPC specialists, who can help implement IPC programs at healthcare facilities, is one key strategy toward sustainable capacity. IPC committees, which may consist of IPC specialists, physicians, nurses, and administrators, can oversee IPC activities and ensure implementation of best practices. IPC guidance must also be tailored to the water, sanitation, and hygiene capacity within healthcare facilities, which is another critical area for improvement.

Despite the apparent resolution of the Ebola epidemic, IPC issues in healthcare facilities will remain critical for a robust healthcare infrastructure. Moving forward, as the international focus shifts to other emerging diseases, it will be important to consider the central role that healthcare facilities play and how existing IPC training tools such as these can be further developed and expanded to address other illnesses. Supporting IPC systems at all levels of the healthcare system will be essential to meaningfully improve country and facility preparedness for future outbreaks.

## Supporting information

S1 Appendix Pre-test / post-test. (DOCX)

S2 Appendix Guinea infection prevention and control training data. (XLSX)
