# Supplementary material for: Infection prevention and control training and capacity building during the Ebola epidemic in Guinea
Source: PLoS One. 2018 Feb 28;13(2):e0193291. doi: 10.1371/journal.pone.0193291 (PMC5831010; doi:10.1371/journal.pone.0193291)
Supplement: S1 Appendix — (DOCX) [file pone.0193291.s001.docx]

**S1 Appendix. PRE-TEST / POST-TEST**

**Introduction à l’Ebola et la prévention et le contrôle de l’infection**

NOM et prénom : ________________________________________________

Date : _________________________

1. Vrai ou faux : Tous les patients atteints d’Ebola ont des saignements des yeux, du nez, et de la bouche.
   1. Vrai
   2. Faux
2. Vous portez les EPI de base (gants, blouse, écran facial, bottes) et le patient devant vous vomit. Que devriez-vous faire ?
   1. Continuer à soigner le patient (vos EPI sont assez)
   2. Quitter la salle d’examen, mettre des EPI complémentaires (deux paires de gants, blouse, écran facial, bottes, bonnet, masque), et retourner soigner le patient
3. Comment est-ce que le virus Ebola se propage? Marquez toutes les réponses correctes.
   1. Par la fumée qui sort des voitures d’OMS et de MSF
   2. Par les moustiques
   3. Par l’eau
   4. Par contact avec les fluides corporels d’une personne atteinte du virus Ebola
4. On jette les aiguilles usagées :
   1. Dans une poubelle
   2. Dans une boîte de sécurité
   3. Dans un sac en plastique
   4. En les posant sur le matelas, à côté du patient
5. Vrai ou faux : Il est possible d’être infecté par le virus Ebola en touchant le corps d’une personne décédée d’Ebola.
   1. Vrai
   2. Faux
6. Vrai ou faux : On peut réutiliser les aiguilles et seringues avec plusieurs patients.
   1. Vrai
   2. Faux
7. Quel est le but du tri ?
   1. Garder les patients hors de l’établissement de soins pour que les médecins et infirmier(e)s n’aient pas autant de travail
   2. Identifier les patients qui pourraient être atteints du virus Ebola et les séparer des patients qui ne sont pas atteints d’Ebola pour éviter la transmission du virus
8. Vrai ou faux : Toucher le vomi, l’urine, ou les selles d’un patient atteint d’Ebola peut vous transmettre le virus Ebola
   1. Vrai
   2. Faux
9. Vrai ou faux : Il faut se laver les mains après avoir enlevé les gants.
   1. Vrai
   2. Faux
10. Vrai ou faux : Les symptômes d’Ebola sont semblables à ceux d’autres maladies, par exemple, le paludisme.
    1. Vrai
    2. Faux
11. Comment pourriez-vous nettoyer le vomi d’un patient ? Marquez toutes les réponses correctes.
    1. Utiliser un balai à franges
    2. Utiliser un chiffon sec
    3. Asperger le vomi avec de l’eau chlorée à 0,05% et essuyer avec un chiffon
    4. Imbiber une serpillère d’une solution chlorée à 0,5% et utiliser la serpillère pour nettoyer le vomi
12. Vous portez les EPI de base (gants, blouse, écran facial, bottes) quand le téléphone sonne. Que devriez-vous faire ?
    1. Répondre au téléphone sans ôter les gants
    2. Ignorer le téléphone
    3. Enlever les gants, répondre au téléphone, et remettre les mêmes gants quand vous avez fini
    4. Enlever les gants, répondre au téléphone, et mettre une nouvelle paire de gants
13. Vrai ou faux : Il y a un médicament qui guéri l’Ebola.
    1. Vrai
    2. Faux
14. Que devriez-vous mettre avant de soigner chaque patient avec la fièvre, l’asthénie, l’anorexie, et la diarrhée dans le contexte de l’épidémie d’Ebola ?
    1. Rien
    2. Seulement un bonnet médical et un tablier
    3. Seulement des bottes, des gants, un masque chirurgical ou une bavette, et un écran facial (si on n’a pas d’écran facial, on peut utiliser des lunettes protectrices)
    4. Seulement une blouse, des gants, un masque chirurgical ou une bavette, des bottes, un bonnet, et un écran facial (si on n’a pas d’écran facial, on peut utiliser des lunettes protectrices)
15. Vrai ou faux : Tout le monde a la responsabilité d’arrêter la propagation du virus Ebola.
    1. Vrai
    2. Faux
16. Lequel des choix suivants vous donnera une solution d’eau chlorée à 0,05% ?
    1. 1 capuchon de Sur’Eau ou Chlore-C dans 20 litres d’eau
    2. 10 cuillères d’HTH à 70% dans 20 litres d’eau
    3. 1 cuillère d’HTH à 70% dans 20 litres d’eau
    4. 4 parties d’eau pour 1 partie d’eau de Javel
17. Vous entrez dans la salle d’examen en portant les EPI de base (gants, blouse, écran facial, bottes). Le patient vomi du sang, et le vomi couvre votre blouse et votre écran facial. Que faites-vous ?
    1. Soignez le patient
    2. Essuyez votre blouse et votre écran facial avec un chiffon imbibé d’eau chlorée à 0,5%
    3. Quittez la salle, enlevez les EPI, mettez les EPI complémentaires (deux paires de gants, blouse, écran facial, bottes, bonnet, masque) et retournez dans la salle pour soigner le patient
18. Quelle concentration d’eau chlorée doit être disponible à l’entrée de l’établissement de soins pour se laver les mains ?
    1. 0,5%
    2. 0,05%
    3. 0,001%
19. Quelle concentration d’eau chlorée doit être utilisée pour désinfecter les surfaces ?
    1. 0,5%
    2. 0,05%
    3. 0,001%
20. Pour la prévention et le contrôle de l’infection dans le contexte de l’épidémie Ebola, chaque établissement de soins doit établir une zone de tri. Quelles personnes doivent passer par la zone de tri en entrant dans l’établissement ?
    1. Seulement les jeunes avec de la fièvre
    2. Seulement les personnes qui ont vomi aujourd’hui
    3. Seulement les personnes qui ont été en contact avec un cas d’Ebola confirmé
    4. Toute personne qui se présente à l’établissement, y compris les agents de soin
21. A combien de mètres de distance du prestataire et des autres patients doit-on faire assoir chaque patient durant le processus de tri ?
    1. 2 mètres
    2. 10 mètres
    3. 5 mètres
    4. 0,5 mètres
22. Avec quelles méthodes devriez-vous prendre la température d’une personne suspectée d’avoir contracté Ebola ? Marquez toutes les réponses correctes.
    1. Avec un thermoflash
    2. Avec un thermomètre mit dans la bouche
    3. Avec un thermomètre axillaire
    4. Avec un thermomètre par voie rectale
23. Les humains peuvent être infectés avec le virus Ebola après avoir touché ou mangé quels animaux ? Marquez toutes les réponses correctes.
    1. Des chauves-souris frugivores infectées avec le virus Ebola
    2. Des animaux sauvages infectés avec le virus Ebola (par exemple : singes, antilopes, porcs épics)
24. On peut être infecté avec le virus Ebola après être entré en contact avec le sang de quelles personnes ? Marquez toutes les réponses correctes.
    1. Personnes malades d’Ebola
    2. Personnes guéries d’Ebola
25. Vrai ou faux : Le risque d’infection avec le virus Ebola est élevé pour les agents de santé, la famille d’un malade d’Ebola, et les personnes qui soignent le malade d’Ebola.
    1. Vrai
    2. Faux
26. Comment les agents de soins peuvent-ils être contaminés avec le virus Ebola ? Marquez toutes les réponses correctes.
    1. En touchant du linge contaminé par le virus
    2. En se piquant accidentellement avec une aiguille utilisée sur un malade d’Ebola
    3. En touchant les fluides corporels d’un patient infecté
    4. En touchant l’époux ou épouse d’un malade d’Ebola, si l’époux ou épouse n’a aucun symptôme
27. Le virus Ebola peut être détruit de quelle façon ? Marquez toutes les réponses correctes.
    1. Avec de l’eau chlorée à 0,05%
    2. Avec de l’eau chlorée à 0,5%
    3. Avec de l’eau savonneuse
    4. En essuyant vigoureusement avec un chiffon sec
28. Un cas probable, un cas suspect, un sujet contact, et un cas confirmé :
    1. Ce sont les mêmes choses
    2. Ce sont des choses différentes
29. Qu’est-ce qu’il faut faire au cours du prélèvement de sang d’un cas suspect d’Ebola ? Marquez toutes les réponses correctes.
    1. Réutiliser les gants avec plusieurs patients
    2. Porter les EPI
    3. Jeter les aiguilles usées sur le sol
30. Lequel des scénarios suivants est une des définitions de cas suspect d’Ebola, pour un patient sans saignements inexpliquées et qui ne sait pas s’il a eu du contact avec un cas d’Ebola ?
    1. Fièvre + 1 symptôme
    2. Fièvre + 2 symptômes
    3. Fièvre + 3 symptômes
